# Fabrication and Application of Novel Porous Scaffold in Situ-Loaded Graphene Oxide and Osteogenic Peptide by Cryogenic 3D Printing for Repairing Critical-Sized Bone Defect

**DOI:** 10.3390/molecules24091669

**Published:** 2019-04-28

**Authors:** Yidi Zhang, Chong Wang, Li Fu, Shan Ye, Min Wang, Yanmin Zhou

**Affiliations:** 1 Department of Oral Implantology, School of Stomatology, Jilin University, Changchun 130021, China; ydzhang16@mails.jlu.edu.cn (Y.Z.); fuli1127@126.com (L.F.); yeshan16@mails.jlu.edu.cn (S.Y.); 2 Jilin Provincial Key Laboratory of Tooth Development and Bone Remodeling, Changchun 130021, China; 3 College of Mechanical Engineering, Dongguan University of Technology, Songshan Lake, Dongguan 511700, Guangdong, China; 4 Department of Mechanical Engineering, The University of Hong Kong, Pokfulam Road, Hong Kong 999077, China; memwang@hku.hk

**Keywords:** osteogenic peptide, sustained delivery, bone tissue engineering, scaffolds, graphene oxide

## Abstract

Osteogenic peptides have been reported as highly effective in directing mesenchymal stem cell osteogenic differentiation in vitro and bone formation in vivo. Therefore, developing novel biomaterials for the controlled delivery of osteogenic peptides in scaffolds without lowering the peptide’s biological activity is highly desirable. To repair a critical-sized bone defect to efficiently achieve personalized bone regeneration, a novel bioactive poly(lactic-*co*-glycolic acid) (PLGA)/β-tricalcium phosphate (β-TCP) composite scaffold, in which graphene oxide (GO) and bone morphogenetic protein (BMP)-2-like peptide were loaded in situ (PTG/P), was produced by an original cryogenic 3D printing method. The scaffolds were mechanically comparable to human cancellous bone and hierarchically porous. The incorporation of GO further improved the scaffold wettability and mechanical strength. The in situ loaded peptides retained a high level of biological activity for an extended time, and the loading of GO in the scaffold further tuned the peptide release so that it was more sustained. Our in vitro study showed that the PTG/P scaffold promoted rat bone marrow-derived mesenchymal stem cell ingrowth into the scaffold and enhanced osteogenic differentiation. Moreover, the in vivo study indicated that the novel PTG/P scaffold with sustained delivery of the peptide could significantly promote bone regeneration in a critical bone defect. Thus, the novel bioactive PTG/P scaffold with a customized shape, improved mechanical strength, sustainable peptide delivery, and excellent osteogenic ability has great potential in bone tissue regeneration.

## 1. Introduction

To date, safely and effectively regenerating bone tissues in a critical-sized defect remains a great challenge; hence, guided bone regeneration (GBR) is required [1,2]. Scaffolds play a critical role in GBR because they provide structural supports, influence cell behaviors, and serve as templates in the process of bone healing [3]. Autografts are the current gold standard because they trigger minimal immunogenic response during the reconstruction, but their applications are restrained by a limited donor supply and secondary trauma. Currently, a highly desirable goal is the production of scaffolds for bone regeneration, as scaffolds can mimic the structure of native bone tissue, release biologically active agents in a controlled way, and degrade with the formation of the new bone. Calcium phosphate-based ceramic materials are favorable for fabricating bone substitutes, as ceramic’s crystal and chemical structures are extremely similar to those of the native bone [4]. However, new bone formation in bioceramic scaffolds alone is limited.

Many clinical studies have demonstrated the excellent osteoinductivity of bone morphogenetic proteins (BMPs), especially BMP-2, but the direct use of BMP-2 in bone regeneration is hindered by its high cost and short half-life [5,6]. Therefore, the BMP-2-like osteogenic peptide, a safe and cost-effective synthetic osteoinductive peptide, has been increasingly used as a potent alternative to induce osteogenesis [7,8]. Generally, the rapid release and degradation of osteoinductive drugs compromise their function. Therefore, the development of a smart delivery system that allows the drug to be released sustainably is necessary for the optimization of osteoinductive drugs [9].

Recently, graphene oxide (GO), a 2D material that is oxidized from graphene and has better solubility and more favorable chemical characteristics than graphene, has been reported to absorb growth factors, collagen, and other proteins [10,11]. Studies have shown that GO can absorb these bioactive substances because it has an ultrahigh surface area-to-volume ratio, covalent conjunction, a π–π stacking structure, and excellent electrostatic interactions with biomolecules [12,13]. On the basis of these binding mechanisms, loading bioactive drugs onto the GO surface is a meaningful strategy to achieve sufficient drug concentrations for an extended time. So far, most studies have incorporated GO or GO-loaded drugs into the scaffolds via post-deposition treatment in light of their unstable properties, but this strategy has proved to be very inefficient [14,15]. Another disadvantage of post-deposition incorporation is that it can cause a relatively high concentration of GO in local areas. A high concentration of GO can generate strong reactive oxygen species (ROS), which are harmful to the tissue repair process [16]. Although GO can be incorporated into scaffolds using electrospinning or a template, it is difficult to achieve the high mechanical strength required for a bone substitute and to match the shape of the bone defect [17,18,19]. Thus, it remains challenging to establish a methodology to produce GO-incorporated scaffolds that can realize the sustained release of bioactive drugs/molecules as well as a controllable shape and microstructure, especially a mechanical strength that matches that of the native bone.

Cryogenic 3D printing is a recently developed rapid prototyping technology that can fabricate scaffolds with a predesigned shape, controllable architecture, and adequate mechanical strength at the relatively low temperature of −32 °C, allowing the incorporation of a large quantity of biomolecules/drugs in situ into scaffolds and the retention of a high level of the biomolecule’s biological activity [20]. Cryogenic 3D printing avoids conventional 3D printing’s disadvantages, such as UV light and the post-sintering use of a high-power laser, and it shows great potential in tissue engineering. To the best of our knowledge, the in situ incorporation of GO-loaded drugs/biomolecules into scaffolds by 3D printing has barely been explored.

In this study, we incorporated GO nanosheets adsorbed with the BMP-2-like osteogenic peptide (GO@peptide) into poly(lactic-*co*-glycolic acid) (PLGA)/β-tricalcium phosphate (β-TCP) composite scaffolds in situ through cryogenic 3D printing for the first time. Our objective is to develop a biodegradable scaffold with improved in vitro osteogenic differentiation capability and enhanced in vivo bone forming ability while avoiding the technical, clinical, and manufacturing limitations of existing 3D-printed bone tissue engineering scaffolds. Our scaffolds, produced through cryogenic 3D printing, were hierarchically porous and mechanically comparable to human cancellous bone. The in situ loading of GO@peptide into scaffolds achieved the sustained release of the peptide, which retained a high level of its biological activity. The proliferation and in vitro osteogenic differentiation of rat mesenchymal stem cells (rMSCs) were also significantly improved by our scaffolds, which also significantly improved the in vivo bone regeneration of cranial defects in rats.

## 2. Materials and Methods

### 2.1. Materials

The BMP-2-like peptide (sequence from N to C: KIPKA SSVPT ELSAI STLYL SGGC) was synthesized by Shanghai ZiYu Biotech Co. (Shanghai, China). GO and β-TCP were purchased from Sigma-Aldrich (St. Louis, MO, USA), and dichloromethane (DCM) was purchased from Aladdin Tech, China. PLGA (LA/GA = 50/50, Mw = 100,000) was obtained from Jinan Daigang Biomaterial Co., Ltd., Jinan, China. Dulbecco’s Modified Eagle Medium (DMEM)/F12 and fetal bovine serum (FBS) were purchased from Gibco, Grand Island, NY, USA. Penicillin–streptomycin and trypsin were obtained from Hyclone (Los Angeles, CA, USA).

### 2.2. Formulation of Water/Oil Composite Emulsion Inks and Cryogenic 3D Printing of Scaffolds

Briefly, 60 mg of GO nanosheets was added to 10 mL of distilled water by ultrasonication for 4 h to obtain a uniform GO/water suspension. Then, 10 mg of the BMP-2-like peptide was dissolved in 300 μL of deionized water to form a peptide/water solution. The BMP-2-like peptide solution was mixed with 1 mL of GO solution and stirred at room temperature for 24 h to allow the absorption of the BMP-2-like peptide onto GO nanosheets. After that, 150 μL of collagen I (COL-I) solution with a concentration of 9.73 mg/mL (Corning, USA) and 3.45 μL of NaOH (1 N) were added to the GO solution and further stirred for 10 min. The GO@peptide solution was successfully produced. Subsequently, 1.5 g of PLGA was dissolved in 10 mL of DCM solution to form a homogeneous solution with a 15% (*w*/*v*) concentration. Afterward, 1 mL of the aforementioned GO@peptide solution and 15 μL of Tween 20 were mixed with 10 mL of the PLGA/DCM solution by using continuous magnetic stirring to form the water/oil emulsion inks. Then, 1.5 g of β-TCP powder was added to the aforementioned ink and stirred with a glass rod to form a uniform liquid paste. A series of PLGA/β-TCP/GO@peptide (PTG) compounds with varied GO concentrations (e.g., 0, 0.025, 0.05, and 0.1 wt.% with respect to the final mixture) was produced and designated as ‘PT/P’, ‘0.025PTG/P’, ‘0.05PTG/P’, and ‘0.1PTG/P’, respectively. The PLGA/β-TCP scaffold without peptide was generated as the negative control and denoted as ‘PT’. The paste was extruded layer by layer at −32 °C from a programmed nozzle to form a cubic porous scaffold with the dimensions 10 × 10 × 5 mm using a cryogenic 3D printing machine (Creality 3D Technology Co., Ltd., Shenzhen, China) according to a predesigned 3D model, in which the macropore size was set to 400 μm, the layer thickness was 0.32 mm, the printing speed was 15 mm/s, the extrusion speed was 0.006 mm/s, and printing was done with a needle of 22G. Afterward, lyophilization was performed for 24 h to remove the DCM.

### 2.3. Characterization of Original Materials, GO Nanosheets and Scaffolds

The phase of GO, β-TCP and PLGA were tested by X-ray diffraction (XRD). High-Performance Liquid Chromatography (HPLC) was used to analyze the purity of the peptides. The morphology and thickness of GO were characterized by atomic force microscopy (AFM, Bruker Dimension Icon, Billerica, MA , USA) under tapping mode. The samples were sputter-coated with 10 nm of gold and then observed under a cold-field emission scanning electron microscope (SEM, SU8010, Hitachi, Japan). The functional groups in the GO and PTG composite scaffolds were analyzed using a fully automated laser Raman microscope (LabRAM HR800, HORIBA Jobin Yvon, France) with a 633 nm excitation wavelength and 50× magnification at room temperature. The static contact angle of water droplets on the scaffolds (*n* = 3 for each group) was determined with an optical measuring instrument. Three different points of a droplet on each sample were measured, and images were captured with a CCD camera (VCA-2000, AST). The porosity of the scaffolds was characterized by the water immersion method. Briefly, the scaffolds were freeze-dried and weighed. Thereafter, the scaffolds were placed in a vacuum oven immersed in water until no bubbles appeared. The porosity (*P*) of the scaffolds was calculated as follows:

(1) $$P=\frac{W_{1}-W_{2}}{W_{1}-W_{3}}\times 100\%$$

where *W*_1_ is the scaffold weight after freeze-drying, *W*_2_ is the weight of the water-saturated scaffold, and *W*_3_ is the floating weight after the scaffold was immersed in water.

### 2.4. In Vitro Peptide Release and Scaffold Degradation

The scaffolds were sectioned into cubes with the dimensions 5 × 5 × 10 mm and then immersed in a 15 mL centrifuge tube containing 10 mL of DMEM/F12 with 1% penicillin–streptomycin at 37 °C for up to 8 weeks in a shaking water bath at 120 rpm; this process was carried out according to the guideline titled Biological Evaluation of Medical Devices—Part 12: Sample Preparation and Reference Materials (ISO 10993-12:2012, URL: https://www.iso.org/standard/53468.html). The degradation medium was changed and collected at specific time points and then subjected to HPLC with a UV–vis detector at a wavelength of 220 nm for detecting the amount of peptide. The scaffolds were lyophilized and subjected to compression testing at week 0, 2, 4, 6, and 8. The compression test was conducted using a universal testing machine (ELF 3200, Bose, USA) at 1 mm/min for 5 min under the standard temperature condition. The weight loss of scaffolds was also examined every 2 weeks for up to 8 weeks. The weight remaining ratio was calculated as follows:

(2) $$\mathrm{Weight}\;\mathrm{remaining}\;\mathrm{ratio}\;(\%)=\frac{W_{t}}{W_{0}}\times 100\%$$

where *W*_0_ is the weight at week 0 and *W*_t_ is the weight tested at different time points after degradation. At least three samples were tested for each type of scaffold, and the mean and standard deviation were calculated.

### 2.5. In Vitro Biological Performance

The rMSCs were isolated from Wistar rats according to established protocols and cultured in DMEM/F12 supplemented with 10% FBS and 1% penicillin–streptomycin at 37 °C in a 5% CO_2_ incubator. Confluent cells were detached with a mixture of 0.25% trypsin. Aliquots of separated cells were then subcultured. The third-passage cells were employed to conduct the following experiment.

#### 2.5.1. Cell Viability Evaluation

The rMSCs were seeded on different scaffolds placed in a 24-well culture plate at a density of 1 × 10^5^ cells/well and incubated at 37 °C for 24 h, allowing the cells to adhere to the scaffolds. After culturing for 1 day, the cell/scaffold constructs were shifted to a new culture plate. The cytotoxicity of the scaffolds was assessed by live/dead assay (Live/Dead Imaging Kit, Molecular Probes, Life Technologies, Waltham, MA, USA) after culturing rMSCs on them for 3 days. In brief, the working solution was prepared by mixing Calcein AM and propidium iodide to stain live and dead cells, respectively. The cells were incubated in the working solution for 15 min and then observed under a fluorescence microscope. The proliferation of rMSCs was detected by the Cell Counting Kit-8 assay (CCK-8, Dojindo Laboratories, Kyushu Island, Japan) on day 1, 4, and 7. Briefly, cells were incubated with blends of CCK-8 solution and fresh medium at a ratio of 1:10 for 1 h, and the OD value of each group was measured at 450 nm with a microplate reader (BioTek, Winooski, VT, USA).

#### 2.5.2. Cell Adhesion and Morphology

The rMSCs were seeded on different scaffolds placed in 24-well culture plates at a density of 1 × 10^5^ cells/well and incubated at 37 °C for 24 h, allowing the cells to adhere to the scaffolds. After culturing for 1 day, the cell/scaffold constructs were shifted to a new culture plate. Cells were fixed with 4% paraformaldehyde for 30 min and washed thrice with PBS. They were added to 200 μL of BSA block solution with 1% (*w*/*v*) concentration and incubated for 1 h. Then, they were incubated with specific primary antibodies (Anti-vinculin, [BM4051, Boster]) overnight at 4 °C, and the cell/scaffold constructs were incubated in an aqueous solution of Alexa Fluor 555 Goat Anti-Rabbit IgG (H + L) (Life Technologies, Waltham, MA, USA) for 1 h. The F-actin of the fixed cells was stained with Alexa Fluor 488 Phalloidin (AAT, Sunnyvale, CA, USA) for 30 min, and 4′,6-diamidino-2-phenylindole (DAPI) was simultaneously added to stain the nuclei for 15 min. The stained cells were observed by confocal laser scanning microscopy (CLSM, FV1000 camera, Olympus Optical, Tokyo, Japan). The densities of F-actin and vinculin were analyzed by measuring the positive F-actin area using Image J software (Bethesda, MD, USA). After culturing for 3 days, the samples were observed by SEM. Cell/scaffold constructs were fixed with 2.5% glutaraldehyde for 12 h, dehydrated by graded ethanol, and dried at the critical point. The samples were sputter-coated with 10 nm of gold before observation.

### 2.6. Osteogenic Differentiation

Approximately 1 g of the scaffolds from each group was immersed in 10 mL of standard osteogenic induction medium containing DMEM/F12 supplemented with 10% FBS, 1% penicillin–streptomycin, 0.25 mM ascorbic acid, 10 mM β-glycerophosphate, and 10 nM dexamethasone. On day 10, the conditioned medium was collected and stored at −20 °C.

#### 2.6.1. ALP Activity and Cell Mineralization

Cells were seeded in a 6-well culture plate at a density of 2 × 10^5^ cells/well, containing the aforementioned conditioned medium. The alkaline phosphatase (ALP) activity of the cells was assessed using the ALP activity colorimetric assay kit (BioVison, Milpitas, CA, USA) and BCA protein kit assay (Thermo Fisher Scientific, Waltham, MA, USA) following the protocol used in our previous investigation [21]. ALP staining (Promega, Madison, WI, USA) was conducted on day 7 to confirm the results of the ALP activity assay. The mineralization of rMSCs was assessed on day 21 by Alizarin Red S (ARS) staining (Solarbio, Beijing, China). Briefly, the cells were fixed with 4% paraformaldehyde for 15 min and stained with 2% Alizarin Red S solution for 30 min at 37 °C, followed by washing with PBS twice. Then, the stained calcium nodules were observed under an optical microscope.

#### 2.6.2. Osteogenic Gene Expression

Quantitative real-time polymerase chain reaction (qRT-PCR) was conducted to determine the osteogenic gene expression of rMSCs on scaffolds. Cells were seeded in a 6-well culture plate at a density of 2 × 10^5^ cells/well and collected after 7 and 14 days. Total RNA was extracted using Trizol reagent (Invitrogen, Waltham, MA, USA) and then reverse-transcribed using the RevertAid kit (Takara Bio, Otsu, Japan) in accordance with the manufacturer’s instructions. Gene expression was assessed using TB Green qPCR Super Mixture (Takara Bio) on the ABI 7300 instrument (ABI, Foster City, Foster City, CA, USA). ALP, Runt-related Transcription Factor 2 (RUNX-2), osteocalcin (OCN), osteopontin (OPN), and COL-I were chosen as target genes for analysis, and glyceraldehyde-3-phosphate dehydrogenase (GADPH) was used as an endogenous housekeeping gene. The sequences of the primers for these genes are given in Table 1 (Invitrogen, USA). The qPCR conditions were as follows: 95 °C for 30 s, followed by 95 °C for 5 s and 60 °C for 31 s for 40 cycles. The relative mRNA expression of ALP, RUNX-2, OCN, OPN, and COL-I was normalized to that of the housekeeping gene.

### 2.7. In Vivo Animal Test

The experimental protocol was formally approved by the Animal Care and Experiment Committee of the Animal Center of Norman Bethune Health Science Center attached to Jilin University (ethics approval number is (2018) No. 53). The in vivo study was pegrformed on 24 male Wistar rats (6 weeks old, body weight: 220–250 g). The rats were randomly divided into 4 groups: the control group rats were left without scaffolds, and the PT, PT/P, and PTG/P groups correspond to rats with scaffolds of differing compositions (n = 6). Briefly, the rats were anesthetized by intraperitoneal injection of chloral hydrate (10%), and then calvarial defects were made using a trephine drill with a diameter of 5 mm on each side of the midline in each animal. The scaffolds, with a diameter of 5 mm and thickness of 1 mm, were implanted in both sides of the animals; then, the soft tissue was carefully sutured. In the control group, the defects were left without scaffolds. Each rat received an intraperitoneal injection of antibiotics for 3 days post-surgery.

#### 2.7.1. Polyfluorochrome Sequential Labeling

Sequential fluorescence labeling was used to evaluate the speed of bone formation. After implantation for 2 weeks and 4 weeks, the rats received alizarin red S (20 mg/kg) and calcein green (20 mg/kg), respectively.

#### 2.7.2. Micro-CT Evaluation and Histological Evaluation

At 4 weeks and 12 weeks post-surgery, the animals were sacrificed; the skulls were removed, together with the bone defect sites, and kept in 10% formaldehyde solution at 4 °C for 10 days. The skulls were scanned using micro-CT (μ-CT50, Scanco, Swiss). Then, the specimens were dehydrated in a graded series of ethanol and soaked in methylmethacrylate polymer. The specimens were cut using Exact Cutting and Grinding Equipment (EXAKT310CP, Oklahoma City, Germany) at the center of the defect with a thickness of 60 μm. Fluorescent labeling was observed under a fluorescence microscope. Then, the sections were stained with toluidine blue staining and methylene blue acid fuchsin staining. Then, the sections were observed under a light microscope (Olympus U-RFL-T, Tokyo, Japan) and analyzed with Image-Pro Plus. In addition, 4 weeks after surgery, the major organs (heart, liver, spleen, and kidney) were collected and fixed with 10% formaldehyde for 24 h. The samples were then dehydrated and embedded in paraffin. The hematoxylin and eosin stain was used to visualize the specimens.

### 2.8. Statistical Analysis

All of the experiments were conducted in quadruplicate. One-way ANOVA followed by the Least Significant Difference (LSD) test and Student–Newman–Keuls (SNK) test were performed to determine the statistical differences using IBM SPSS 23.0. For all analyses, *p* < 0.05 (*) indicates statistically significant differences.

## 3. Results

### 3.1. Design of PTG/P Scaffolds

**Figure 1:**
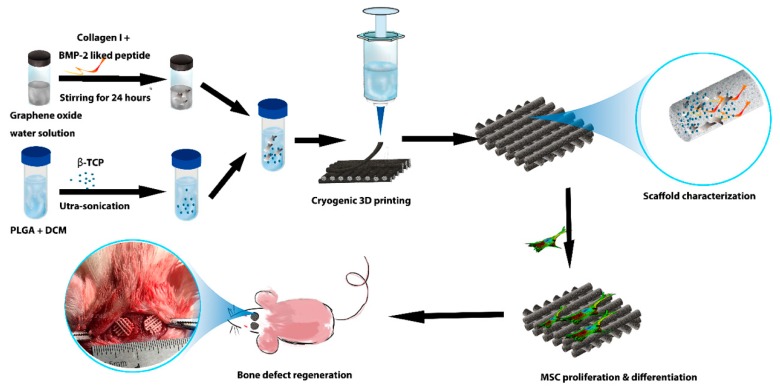
Schematic for the fabrication and function of peptide/GO/β-TCP/PLGA scaffolds.

Figure 1 shows the schematic of the production and function of the PTG/P scaffolds. The peptide and COL-I were first adsorbed onto the GO nanosheets and then loaded into PLGA/β-TCP/DCM solutions to form the composite emulsions used as printing inks. The collagen solution and the DI water added to the mixture protected the biological activity of the peptides from the organic solution. Afterward, PTG/P scaffolds with a designed shape and macroscopic architecture could be built via cryogenic 3D printing. Our design allows for a high loading level of the peptide. The in vitro rMSC osteogenic differentiation and in vivo bone regeneration were achieved via the controlled delivery of the peptide and the unique morphology of the scaffolds. In addition, the functional groups in GO can interact with the amino groups in the peptide, and the interaction also helps control the release rate of peptides. This design is highly favorable for the proliferation and osteogenic differentiation of rMSCs.

### 3.2. Characterization of Scaffolds

**Figure 2:**
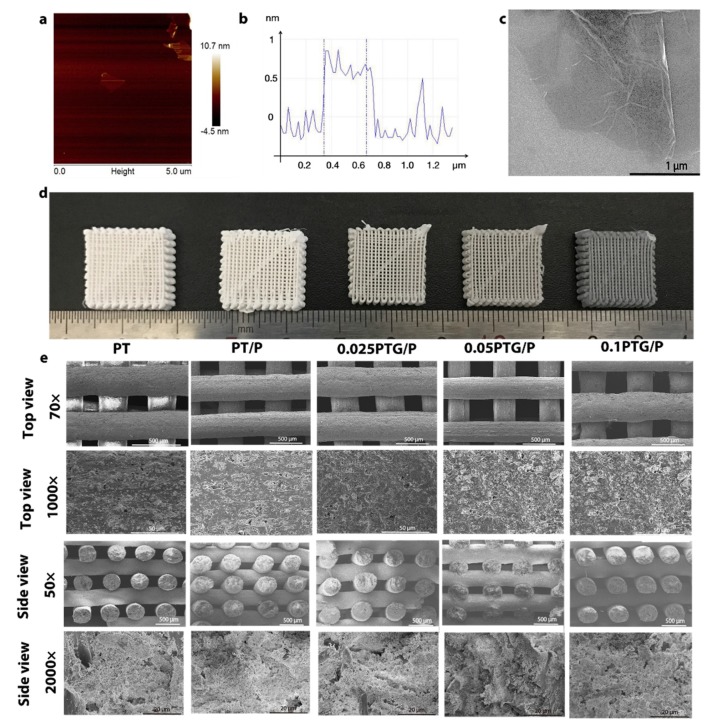
Characterization of the scaffolds: (**a**) AFM topography images of GO; (**b**) Analysis of the height profiles of GO; (**c**) SEM images of GO; (**d**) Images of PT, PT/P, 0,025PTG/P, 0.05PTG/P, and 0.1PTG/P (from left to right); (**e**) SEM and high-resolution SEM images of PT, PT/P, 0,025PTG/P, 0.05PTG/P, and 0.1PTG/P.

The XRD spectrum and HPLC results of peptide are shown in Figure S1. The AFM images of GO nanosheets suggest that GO nanosheets with an average height of 0.5–1 nm had a single-layer structure (Figure 2a,b). The morphology of GO nanosheets, as detected by SEM, is shown in Figure 2c. The cryogenic 3D-printed scaffolds had a well-designed latticed structure with the dimensions 15 × 15 × 5 mm (Figure 2d). Uniform square macropores with an average pore size of 400 ± 50 μm were observed in all scaffolds, and no statistical difference was found among these groups (Figure 2e). Numerous micropores with a 5–50 μm diameter were observed on the strut surface. This could be attributed to the ice particles formed in the frozen PLGA/β-TCP pattern, which maintained the space until freeze-drying. The colors of these scaffolds changed from white to black with the increase of GO content, which could be attributed to the influence of the natural color of GO. Figure S2 shows the Raman spectrum mapping of the D band density of PTG scaffold, indicating that the GO is uniformly distributed on the surface of PTG/P scaffold. Figure S3 shows that the GO nanosheet without peptide showed a smooth morphology (Figure S3a). After adding the peptide, the morphology became rippled with homogeneous wrinkles (Figure S3b).

**Figure 3:**
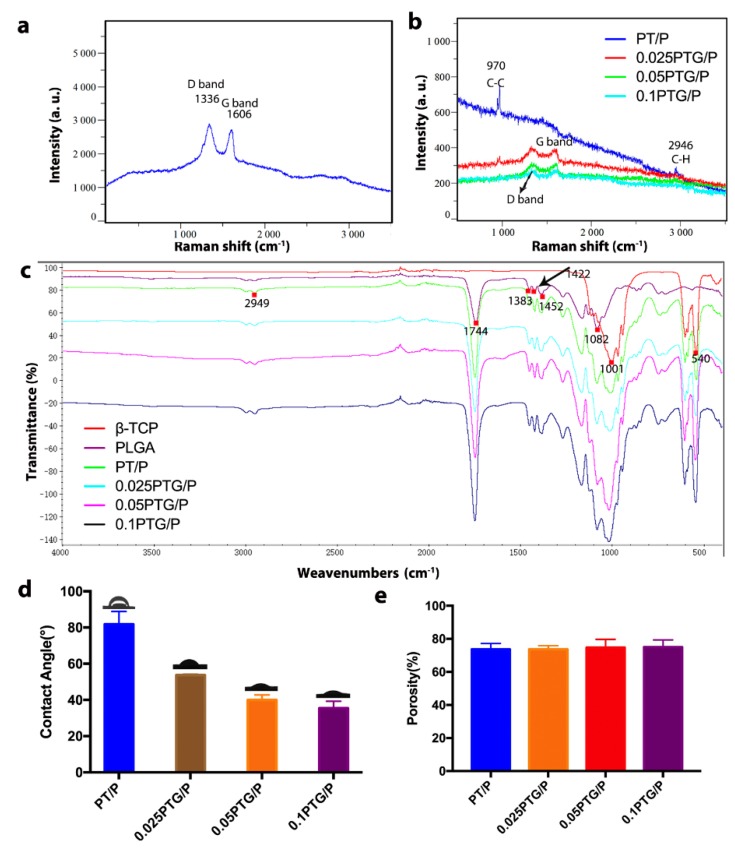
Characterization of surface chemical properties: (**a**) Raman spectrum of GO; (**b**) Raman spectra of PT/P, 0.025PTG/P, 0.05PTG/P, and 0.1PTG/P; (**c**) FTIR-ATR spectra of scaffolds; (**d**) WCA of PT/P, 0.025PTG/P, 0.05PTG/P, and 0.1PTG/P; (**e**) Porosity of PT/P, 0.025PTG/P, 0.05PTG/P, and 0.1PTG/P.

The Raman spectrum shows how the GO nanosheets interacted with the PT scaffolds. The spectrum of GO shows two characteristic peaks of the D and G bands at 1336 and 1606 cm^−1^, respectively (Figure 3a). In the spectrum of PT scaffolds, peaks at 2946 cm^−1^ were attributed to the stretching vibration of C–H. Peaks at 970 cm^−1^ were due to the stretching vibration of C–C. No prominent bands within 1300–1600 cm^−1^ were observed in the PT scaffold (Figure 3b). In the spectra of the 0.025PTG/P, 0.05PTG/P, and 0.1PTG/P scaffolds, the D and G band peaks were observed, indicating the successful hybridization of GO and the PT/P scaffold. Figure 3c shows the FTIR-ATR spectra of PLGA, β-TCP, PT/P, 0.025PTG/P, 0.05PTG/P, and 0.1PTG/P scaffolds. For β-TCP, the peaks at 1001 cm^−1^ could be the PO_4_^3−^ group in β-TCP, and the peaks at around 540 cm^−1^ could be Ca and P particles in β-TCP. For PLGA, the peaks at 2949 cm^−1^ could be the stretching vibrations of C–H, and the peaks at 1452, 1422, and 1383 cm^−1^ could be the bending vibration of C–H. The peaks at 1744 and 1082 cm^−1^ could be the stretching vibration of C=O and C–O, respectively. The spectra of PT/P, 0.025PTG/P, 0.05PTG/P, and 0.1PTG/P show both of these peaks, indicating successful integration. Figure 3d shows the water contact angles (WCAs) of different scaffolds. PT scaffolds had a relatively high average WCA of 81.75 ± 7.13°. However, for the PTG/P groups, the average WCA decreased from 53.7 ± 0.26° to 35.48 ± 3.84° with increasing GO content. The porosity of the scaffolds was approximately 75%, and no statistical difference was found among the different groups (Figure 3e).

### 3.3. Scaffold Degradation and In Vitro Peptide Release

**Figure 4:**
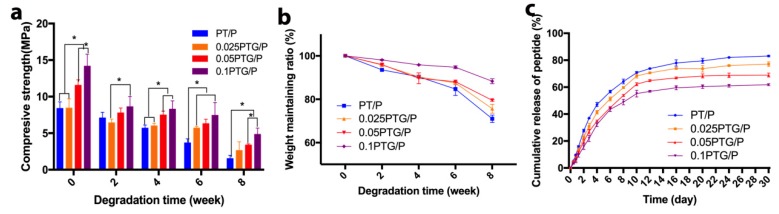
Degradation performance and peptide release of different scaffolds: (**a**) Compressive strength changes in PT/P, 0.025PTG/P, 0.05PTG/P, and 0.1PTG/P scaffolds during degradation; (**b**) Weight remaining ratio of PT/P, 0.025PTG/P, 0.05PTG/P, and 0.1PTG/P scaffolds; (**c**) Release of BMP-2-like peptide from PT/P, 0.025PTG/P, 0.05PTG/P, and 0.1PTG/P scaffolds during degradation. * *p* < 0.05.

To investigate the effects of GO nanosheets on the degradation behavior of PT/P composite scaffolds, the weight loss and compressive strength were measured. The average compressive strength of PT/P, 0.025PT/P, 0.05PT/P, and 0.1PT/P scaffolds at week 0 was 8.39 ± 0.87, 8.44 ± 1.25, 11.56 ± 0.749, and 14.17 ± 1.63 MPa, respectively, suggesting that these scaffolds had enough strength to withstand the external forces during the regeneration process of the non-load-bearing bone tissue. Notably, scaffolds with GO incorporated presented higher compressive strength than that of the PT and PT/P groups. During the degradation test period, the 0.05PTG/P and 0.1PTG/P groups showed a higher compressive strength compared with the PT/P and 0.025PTG/P groups. The decrease in compressive strength became significant after week 6 (Figure 4a). Figure 4b shows that the weight of all scaffolds changed slightly in the first 2 weeks. From 6 weeks to 8 weeks, significant weight changes were obtained for all scaffolds except the 0.1PTG/P scaffolds. Slower weight loss was observed in the 0.05PTG/P and 0.1PTG/P groups. The overall morphology of the scaffolds did not change significantly. However, more pores with bigger size were observed in SEM images (Figure S4). The release of the peptide from the 0.025PTG/P, 0.05PTG/P, and 0.1PTG/P scaffolds was slower than that from the PT/P scaffolds (Figure 4c). The peptide release from the 0.05PTG/P scaffolds plateaued after 12 days. No significant difference was observed between the 0.025PTG/P and the PT/P groups. The 0.1PTG/P scaffolds had the lowest peptide release rate, in which only 61.82 ± 0.68% of the peptide was released within 30 days.

### 3.4. In Vitro Biological Performance

#### 3.4.1. Cell Viability

**Figure 5:**
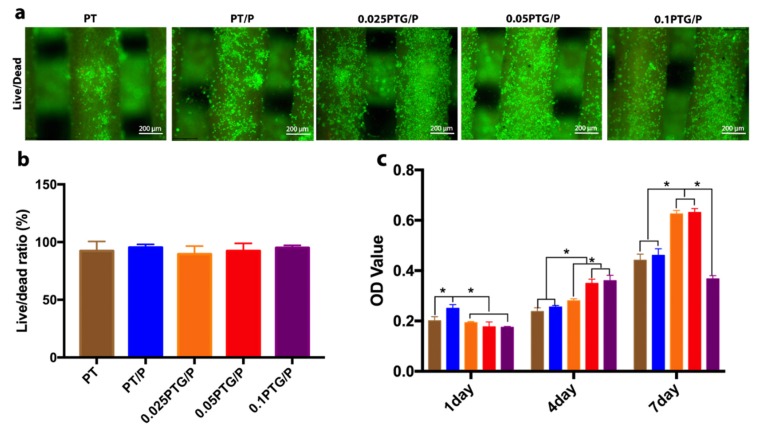
Cell viability and cell proliferation: (**a**) Fluorescence micrographs representing the live (green) and dead (red) cells of rMSCs cultured on PT, PT/P, 0.025PTG/P, 0.05PTG/P, and 0.1PTG/P scaffolds after being cultured for 1 day; (**b**) Live cell/dead cell ratio from the live/dead assay; (**c**) Cell proliferation of rMSCs cultured on PT, PT/P, 0.025PTG/P, 0.05PTG/P, and 0.1PTG/P scaffolds after being cultured for 1, 4, and 7 days. * *p* < 0.05.

The cytotoxicity of the scaffolds was examined by live (green)/dead (red) assay after culturing the rMSCs on the scaffolds for 3 days. The rMSCs displayed a good initial adhesion among all the groups, and there was no dead cell detected on the scaffolds (Figure 5a). The average live cell ratio on the scaffolds was approximately 97.8%, and no statistical difference in the live cell ratio was found among the different scaffolds (Figure 5b).

Cell proliferation was examined on days 1, 4, and 7 using the CCK-8 kit assay. The absorbance of rMSCs cultured on all scaffolds showed an increase in the proliferation rate with increasing culture time (Figure 5c). The proliferation rate of the PT/P group was observed to be higher than the other groups on the first day, possibly because of the fast release of the peptide. The 0.025PTG/P and 0.05PTG/P scaffolds showed a significant increase in proliferation rate from day 4 to day 7, and the increase was greater than that of the other groups. The rMSCs on the 0.1PTG/P and PT scaffolds proliferated slower than those in the other groups as measured on day 7 (*p* < 0.05).

#### 3.4.2. Cell Adhesion and Morphology

**Figure 6:**
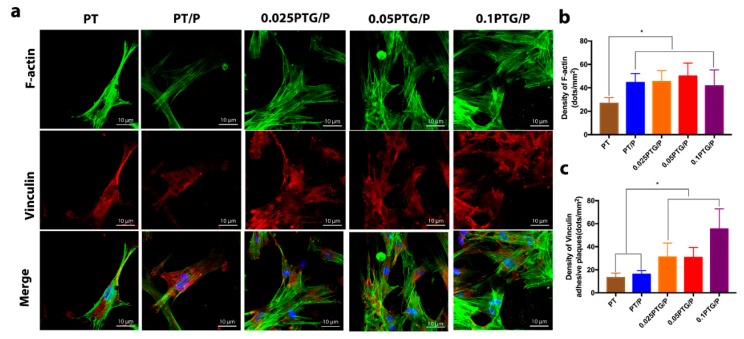
CLSM images of rMSCs on the different scaffolds: (**a**) CLSM images show the cytoskeleton and adhesive plaques of rMSCs on PT, PT/P, 0.025PTG/P, 0.05PTG/P, and 0.1PTG/P scaffolds, with F-actin stained in green, nuclei stained by DAPI, and vinculin stained in red after cells were cultured on scaffolds for 1 day; (**b**) Relative fluorescence unit analysis of F-actin in different groups; (**c**) Relative fluorescence unit analysis of vinculin in different groups. * *p* < 0.05.

Figure 6 shows the CLSM images of the cytoskeleton and adhesive plaques of rMSCs cultured on different scaffolds for 24 h. The fluorescence micrographs clearly reveal that rMSCs had successfully attached to the scaffolds and spread well. Evident F-actin filaments were observed in all groups (Figure 6a). Extensive F-actin filaments and more vinculin adhesive plaques were expressed in rMSCs on the 0.025PTG/P, 0.05 PTG/P, and 0.1PTG/P scaffolds compared with those on the PT and PT/P scaffolds, indicating good initial adhesion of rMSCs (Figure 6b,c). These results suggest that the incorporation of GO into the scaffolds positively influenced the adhesion and maturation of rMSCs.

**Figure 7:**
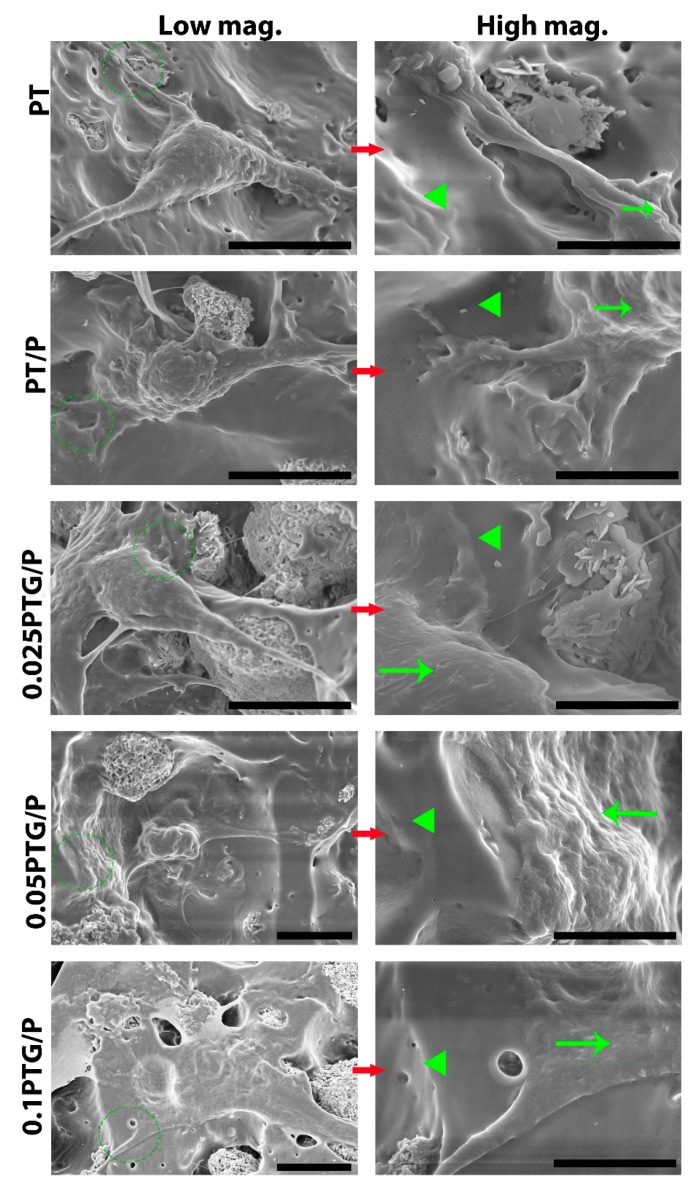
SEM images show the morphology of rMSCs on different scaffolds with low magnification and high magnification of the green circles in the corresponding low magnification images. Notes: green triangles represents the PLGA matrix; the green arrows represent the cell membrane; scale bars of low magnification images are 10 μm; scale bars of high magnification images are 5 μm.

SEM images show that the morphologies of rMSCs on the 0.025PTG/P, 0.05PTG/P, and 0.1PTG/P scaffolds were plumper than those on the PT scaffolds, exhibiting a typical osteoblast-like morphology. The high magnification images clearly showed the boundary between cells and the scaffolds. The cell membrane was rougher than the surface of the scaffolds (Figure 7).

### 3.5. Osteogenic Differentiation of rMSCs

**Figure 8:**
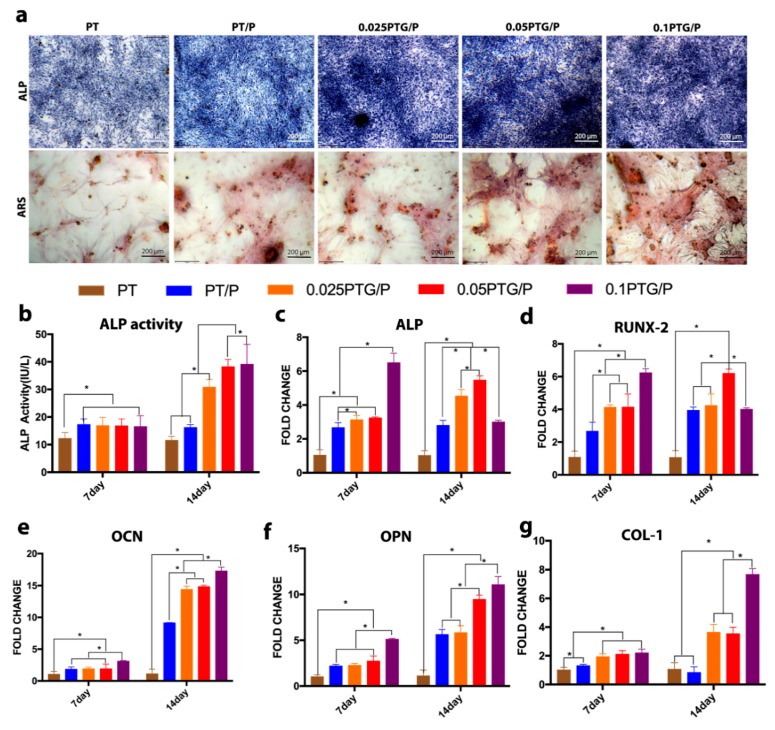
In vitro osteogenic differentiation of rMSCs: (**a**) ALP staining after 7-day induction and ARS staining after 21-day induction. (**b**) ALP activity determined by ALP activity assay. Relative gene expression of (**c**) ALP, (**d**) RUNX-2, (**e**) OCN, (**f**) OPN, and (**g**) COL-I. *p < 0.05.

ALP staining was conducted to study the osteogenic differentiation of rMSCs. Compared with those in the PT and PT/P groups, the rMSCs in the 0.025PTG/P, 0.05PTG/P and 0.1PTG/P groups were larger and darker in color (Figure 8a), suggesting that the addition of GO enhanced the ALP expression of rMSCs; this observation was further confirmed by the ALP activity assay (Figure 8b). The cells cultured on the PT/P scaffolds had higher ALP activity than those in the PT group on day 7. On day 14, the scaffolds containing GO displayed an obvious increase in ALP activity, and the 0.05PTG/P and 0.1PTG/P groups showed the highest ALP activity among all the groups (*p* < 0.05). ARS staining was used to examine the mineralization of rMSCs after being treated with the conditioned medium for 21 days. Obvious calcium nodules were observed in the peptide-containing groups, whereas no discernible calcium nodule was present in the PT group (Figure 8a). Compared with those of the other groups, the calcium nodules deposited in the 0.05PTG/P and 0.1PTG/P groups were larger. Furthermore, no difference between the 0.025PTG/P and PT/P groups was found.

The qPCR results showed the gene expression levels of bone-related genes, including ALP, RUNX-2, OCN, OPN, and COL-I, of rMSCs. For ALP, the 0.1PTG/P group had the highest gene expression level on day 7, but this level had decreased by day 14 (Figure 8c). The ALP expression in the 0.025PTG/P and 0.05PTG/P groups was significantly upregulated on day 14, and the levels of expression were higher than those of the other groups (*p* < 0.05). For RUNX-2, the expression was upregulated with increasing GO content on day 7. In comparison, on day 14, the expression level of the 0.1PTG/P group had significantly decreased (*p* < 0.05), and that of the 0.05PTG/P group was the highest. No significant change was observed in the 0.025PTG/P, PT/P, and PT groups from day 7 to day 14 (Figure 8d). For OCN and OPN, the expression levels of the PT/P, 0.025PTG/P, 0.05PTG/P, and 0.1PTG/P groups were higher than those of the PT group on day 7. Significant upregulation was observed in the PT/P, 0.025PTG/P, 0.05PTG/P, and 0.1PTG/P groups on day 14, and a higher expression level in the GO-containing groups was observed compared with that in the PT/P group (*p* < 0.05) (Figure 8e,f). The expression level of COL-I is shown in Figure 8g. The expression level was higher in the 0.025PTG/P, 0.05PTG/P, and 0.1PTG/P groups than in the PT and PT/P groups on day 7. This trend was maintained until day 14, at which time the 0.1PTG/P group exhibited the highest level (*p* < 0.05).

### 3.6. In Vivo Bone Regeneration Ability

**Figure 9:**
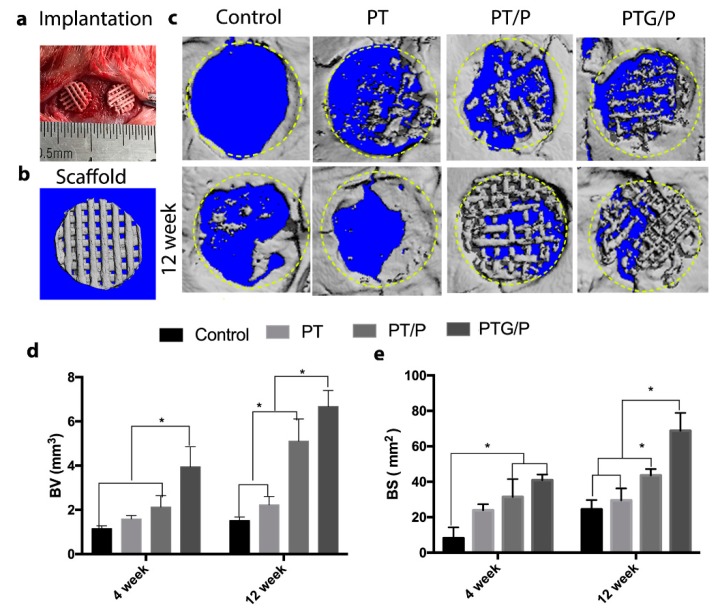
The micro-CT results of the in vivo animal test: (**a**) Implantation procedure; (**b**) Micro-CT image of the scaffold; (**c**) 3D reconstructed images of the control, PT, PT/P, and PTG/P scaffolds after implantation at 4 weeks (top row) and 12 weeks (bottom row); (**d**) The bone volume (BV) of the newly formed bone in the critical-size defects; (**e**) The bone surface (BS) of the newly formed bone in the critical-size defects. * *p* < 0.05.

To further study biological performance, we conducted an in vivo animal test for 12 weeks to confirm the bone regeneration ability of the scaffolds. Critical-sized calvarial defects were successfully created in rats to establish the animal model (Figure 9a). Scaffolds with a diameter of 5 mm were implanted in the animal models to evaluate in vivo bone regeneration. The 3D reconstructed images are displayed in Figure 9c. At 4 weeks, little bone was formed in the control group, while, for the implant groups, more bone was formed in the scaffolds with peptide compared with the PT group. No significant difference was observed between the PT/P and PTG/P groups at week 4. When the implantation time was extended to 12 weeks, more bone was observed in the groups with scaffolds than the control group. Notably, there was more bone in the PTG/P group compared with the other groups. Furthermore, the bone volume (BV) and bone surface (BS) were quantitatively analyzed to confirm the bone regeneration ability. The BV results showed that the animals in the PTG/P group formed more bone than those in the other groups (Figure 9d). The BS results showed the same trend as that observed for BV (Figure 9e).

**Figure 10:**
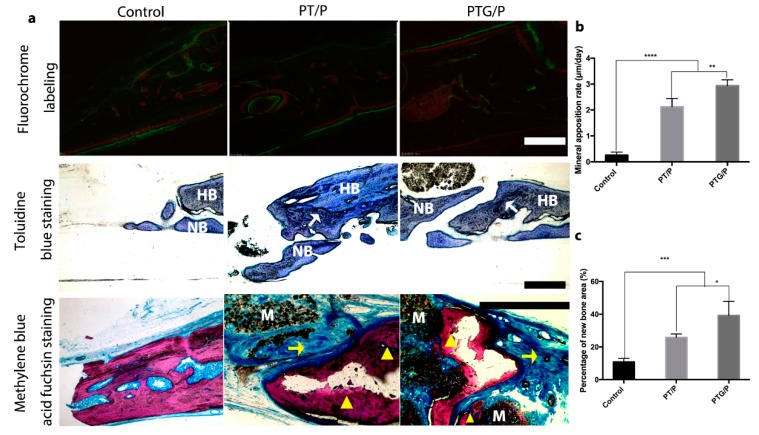
Bone regeneration after 12 weeks: (**a**) Sequential labeling at 2 weeks (alizarin red S) and 4 weeks (green calcein); Toluidine blue staining of calvarial bone at 12 weeks after surgery. “HB” represents host bone; “NB” represents newly formed bone. There is a line between newly formed bone and the host bone (white arrow); Methylene blue acid fuchsin staining of calvarial bone at 12 weeks after surgery. Notes: yellow triangles indicate the new bone and yellow arrows represent osteoid bone. “M” represents the residual materials. (**b**) Mineral apposition rate from 2 weeks to 4 weeks post-surgery; (**c**) Percentage of new bone area (%). Scale bars: 250 μm. * *p* < 0.05, ** *p* < 0.01, *** *p* < 0.001, **** *p* < 0.0001.

We used the fluorescent labeling technique to assess the speed of bone formation. In Figure 10a, there are more red alizarin and green calcein labels in the PT/P and PTG/P groups than there are in the control group; the labels reflect new bone formation at 2 and 4 weeks post-surgery. We also analyzed the mineral apposition rate (MAR), and Figure 10b shows that the MAR was significantly higher in the PTG/P group (2.94 ± 0.23 μm/day) than the PT/P group (1.62 ± 0.38 μm/day). Furthermore, toluidine blue staining revealed that there was a line between the host bone and the native bone (white arrow in the figure), and more new bone was formed in the PTG/P group (Figure 10a). In the images of Methylene blue acid fuchsin staining, new bone formation (dark pink staining) is evident around the residual PTG/P scaffolds. In the control group, there was a slight amount of new bone formed at the edges of the host bone (pink staining), with more osteoid bone (blue staining) present (Figure 10a). Quantitative analysis of new bone formation (Figure 10c) indicated that there was more new bone formation in the PTG/P group than that in the other groups, confirming that the PTG/P scaffold could significantly promote bone regeneration in vivo. In addition, no abnormalities were detected in pathological sections of major organs (Figure S5).

## 4. Discussion

The primary objective of this study is to produce a biodegradable composite scaffold with significantly improved bone forming ability. This study is the first to realize this objective by the in situ loading of an osteogenic peptide and GO nanosheets into non-sintered scaffolds through advanced cryogenic 3D printing. Compared with scaffolds made by conventional 3D printing techniques, our scaffolds not only have a biomimetic macro/microstructure but also are mechanically similar to human cancellous bone, which is desirable for a bone regeneration substitute. Moreover, our scaffolds could load a significantly higher level of the osteogenic peptide and GO via in situ incorporation without lowering the biological activity of the peptide. GO was also found to further tune the release behavior of the peptide to be more sustained. Such sustained delivery of the osteogenic peptide with both high biological activity and high loading level was expected to significantly improve in vitro osteogenic differentiation and in vivo bone regeneration.

The surface morphology of the scaffolds can influence cell adhesion, proliferation, and even differentiation [22]. Porous scaffolds with a macropore size of 100–500 μm have been shown to facilitate nutrient transport, creeping substitution of bone, and vascular ingrowth [23,24,25]. In addition, the microstructures on the surface of the scaffolds successfully extended the surface area and improved the roughness of the scaffolds, both of which are favorable for initial cell attachment, adhesion, and spreading [26,27,28]. In our study, the rectangular macropores had a side length of 400 ± 50 μm, and the micropores on struts had a diameter of 5–50 μm, which are dimensions that meet the standards cited above. Different from other studies, the cryogenic 3D printing method created a hierarchical structure in an environmentally friendly manner by using water droplets in emulsion inks as the pore-forming agent of micropores. The cryogenic environment turns the water droplets into ice particles and reserves them in the scaffold, resulting in a hierarchically porous structure with macro- and microfeatures after lyophilization. Additionally, according to the Raman spectra and FTIR results, the GO nanosheets were successfully incorporated into the scaffolds, and they not only endowed the scaffolds with a specific nanostructure but also enhanced their wettability by introducing COOH^-^ and OH^-^ to the scaffolds’ surface.

An ideal scaffold for bone regeneration serves as a template during the bone regeneration process and is replaced by the newly formed bone as it degrades. Good mechanical performance is of great importance because it allows the scaffolds to maintain their space, withstand external forces, and avoid stress shielding. Volatilization of the organic solvent during the fabrication process would cause deformation of the scaffolds and thus reduce the mechanical strength. The low temperature of cryogenic 3D printing used in this study prevented the deformation of the scaffolds, endowing them with high mechanical strength. Besides this, we found that the incorporation of GO significantly improved the compressive strength of the PTG/P scaffolds as a result of its excellent physical-chemical properties [29,30]. Notably, the compressive strength of the 0.025PTG/P, 0.05PTG/P, and 0.1PTG/P scaffolds was within the range of that of trabecular bone (2–20 MPa) [31] after degrading for 8 weeks; thus, the scaffold conferred temporary support and guaranteed its function during the mineralization process [29,30,31,32]. The weight loss was also influenced by the incorporation of GO. A slower weight change of the PTG/P composite scaffolds compared with that of the PT/P scaffolds could imply that the interactions between GO nanosheets and the PT/P matrix stabilized the matrix, thus reducing the degradation rate [29].

Cryogenic 3D printing resulted in the in situ loading of the peptide and GO, allowing the peptide to diffuse into the scaffolds and be released from a deeper layer of the scaffold matrix to the outer environment through the pores formed by scaffold degradation, and this could slow the sustained release rate of the peptide in the scaffolds. The functional groups, such as epoxide, carboxyl, and hydroxyl groups, that are present on the basal plane and edges of GO can interact with peptides through covalent, electrostatic, and hydrogen bonding [33], further explaining the slower peptide release in the PTG/P scaffolds compared with that of the PT/P scaffolds. These results indicate that our PTG/P scaffolds can serve as an effective vehicle system for sustained peptide delivery, which is conducive to the favorable biological performance of the scaffolds.

Bone regeneration materials should stimulate favorable cellular responses [34]. The macro/micro/nanosurface, as well as the enhanced wettability of our scaffolds, significantly improved cell attachment. In addition, the interconnective pores in the scaffolds would facilitate nutrient transportation and metabolic waste removal, thus making it a friendly microenvironment in which rMSCs can proliferate and infiltrate. The cytotoxicity of GO nanosheets has been reported to be associated with their size, concentration, and interaction time; that is, GO with a larger size and a higher local concentration will induce a decrease in cell viability, while GO with a lower concentration and a smaller size can positively affect cellular behaviors [35]. Cryogenic 3D printing formed a macro/micro/nanostructure in a green process and retained the bioactivity as well as the original properties of the raw materials, which can minimize the cytotoxicity of the scaffolds and release the biomolecules/drugs in a long-term process. According to the degradation results, further controlling GO enabled the PTG/P scaffolds to release the peptide more sustainably, which was favorable for the growth and osteogenic differentiation of rMSCs. Therefore, the sustained release of a bioactive factor could explain the increase in cell proliferation on the 0.025PTG/P and 0.05PTG/P scaffolds on day 4 and day 7. The slower proliferation rate for the 0.1PTG/P group observed on day 7 compared with the 0.025PTG/P and 0.05PTG/P groups may be due to the high concentration of GO generating ROS, which compromises the proliferation rate. Vinculin and F-actin are mainly expressed during the formation of focal adhesion and the aggregation assembly of membrane cytoskeletons, respectively [36]. F-actin and vinculin could reflect the cytoskeleton and adhesion plaques of cells. The low F-actin and vinculin density of PT group was due to the contracted cell morphology and the low cell viability according to the CCK-8 results. The high F-actin density of PT/P, 0.025PTG/P, 0.05PTG/P and 0.1PTG/P groups was due to the rapid peptide release in the initial stage which was favorable for the cell growth. The vinculin density was similar for PT and PT/P group. The higher vinculin density of 0.025PTG/P, 0.05PTG/P and 0.1PTG/P groups compared to PT and PT/P group was because that the oxygen-containing group and topographical features of GO contained scaffolds could greatly enhance the initial cell adhesion [37,38]. In addition, the higher wettability of these GO contained scaffolds could also explain the higher vinculin density of rMSCs on them [39]. Moreover, GO was reported to adsorb serum protein and pro-concentrate osteogenic inducers, such as dexamethasone, β-glycerophosphate, and ascorbic acid, through the interaction between the π–π electron cloud in GO and the aromatic rings in the biomolecules, which is also beneficial for osteogenic differentiation [34,35,36,37,38,39,40]. Therefore, the increase in ALP activity level in the 0.05PTG/P and 0.1PTG/P groups on day 14 may be the result of the sustained release of the peptide and the intrinsic properties of GO. In addition, the improved cell mineralization also suggests the excellent osteostimulatory activity of the PTG/P scaffolds. The osteogenic differentiation ability was examined at the gene expression level. ALP and RUNX-2 are expressed at the early stage of bone formation. The former is related to active bone formation, whereas the latter is an important transcription factor for osteogenic differentiation, and it regulates the downstream expression of other phenotypic markers [41,42,43]. In comparison, OCN and OPN are expressed at the late stage of osteogenic differentiation and are associated with the formation of mineralized bone-like nodules [44]. In this study, the notable upregulated expression of the ALP and RUNX-2 genes in the 0.1PTG/P group could be attributed to the expression of these two genes at the early stage of bone formation, and the high GO concentration could accelerate their expression and cell maturation at an early stage [18]. In accordance with the cell mineralization results, the sustained release of the peptide also significantly increased the expression level of OCN, OPN, and COL-1 measured on day 14. In our in vivo study, the scaffolds containing GO showed better bone formation ability than the PT/P group after 12 weeks; this finding could be attributed to the ability of the scaffolds containing GO to release the peptide in a more sustained manner than the PT/P scaffolds. The sustained release of the peptide constantly stimulates bone regeneration in vivo; hence, more bone was formed around the PTG/P scaffolds. In summary, the sustained release of the peptide and the incorporation of GO nanosheets into the scaffolds significantly upregulated the osteogenic differentiation of rMSCs and could achieve long-term bone regeneration.

## 5. Conclusions

In this study, PTG/P composite scaffolds with in situ loading of GO@peptide were successfully produced using cryogenic 3D printing. The scaffolds had a hierarchically porous structure with controlled macro- and micropores. The compressive strength of the scaffolds improved with increasing GO content, which also enhanced their surface wettability. The sustained release of the peptide in the PTG/P scaffolds due to the in situ loading strategy and the high adsorption ability of GO nanosheets was beneficial for bone growth and promoted the proliferation and adhesion of rMSCs. Additionally, the incorporation of GO nanosheets into the scaffolds significantly enhanced the osteogenic differentiation of rMSCs. Finally, the PTG/P scaffolds significantly improved bone regeneration in vivo. These results indicate that the cryogenic 3D-printed PTG/P scaffolds are a promising platform for inducing bone regeneration.

## Figures and Tables

**Table 1:** Forward (F) and reverse (R) primers for target genes.

Primer	Sequence (5ʹ–3ʹ) (Forward)	Sequence (5ʹ–3ʹ) (Reverse)
GADPH	ACCACAGTCCATGCCATCAC	TCCACCACCCTGTTGCTGTA
ALP	GTCCCACAAGAGCCCACAAT	CAACGGCAGAGCCAGGAAT
RUNX-2	CCACCTCTGACTTCTGCCTC	TATGGAGTGCTGCTGGTCTG
OCN	GGGCAATAAGGTAGTGAA	GTAGATGCGTTTGTAGGC
OPN	AGCTGGATGACCAGAGTGCT	TGAAATTCATGGCTGTGGAA
COL-I	CCTGGAAGAGATGGTGCT	CATTCTTGCCAGCAGGAC

## Supplementary Materials

The following are available online at https://www.mdpi.com/1420-3049/24/9/1669/s1.
