# Supplementary material for: Fabrication and Application of Novel Porous Scaffold in Situ-Loaded Graphene Oxide and Osteogenic Peptide by Cryogenic 3D Printing for Repairing Critical-Sized Bone Defect
Source: Molecules. 2019 Apr 28;24(9):1669. doi: 10.3390/molecules24091669 (PMC6539066; doi:10.3390/molecules24091669)
Supplement: Supplementary file 1 [file molecules-24-01669-s001.pdf]

Supplementary information

Manuscript ID: Molecules-472929

Fabrication and application of novel porous scaffold *in situ* loaded graphene oxide and osteogenic peptide by cryogenic 3D printing for repairing critical-sized bone defect

Yidi Zhang [1], Chong Wang [2, *], Li Fu[1], Shan Ye [1] Min Wang[3], and Yanmin Zhou [1, *]

[1] Department of Oral Implantology, School of Stomatology, Jilin University, Changchun, China.; Jilin Provincial Key Laboratory of Tooth Development and Bone Remodeling, Changchun 130021, China; ydzhang16@mails.jlu.edu.cn (Y. Z.); fuli1127@126.com; yeshan16@mails.jlu.edu.cn (S. Y.);
[2] College of Mechanical Engineering, Dongguan University of Technology, Songshan Lake, Dongguan, Guangdong 511700, China;
[3] Department of Mechanical Engineering, The University of Hong Kong, Pokfulam Road, Hong Kong SAR, China; memwang@hku.hk
* Correspondence: wangchong@dgut.edu.cn (C. W.); zhouym@jlu.edu.cn (Y. Z.); Tel: 86-134-1688-5162 (C. W.); 86-0431-8879-6025 (Y. Z.)

This PDF file includes:

Figure S1-S5.

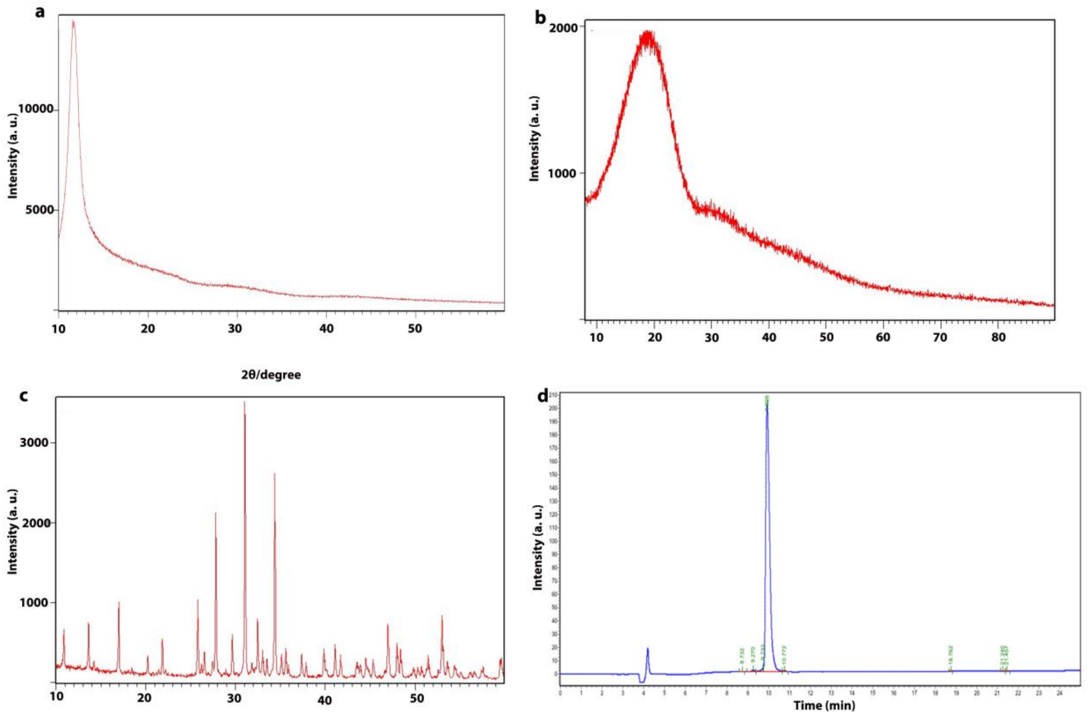

Figure S1. materials characterization: XRD patterns of (a) GO; (b) PLGA; (c) β-TCP. (d) HPLC results of peptide.

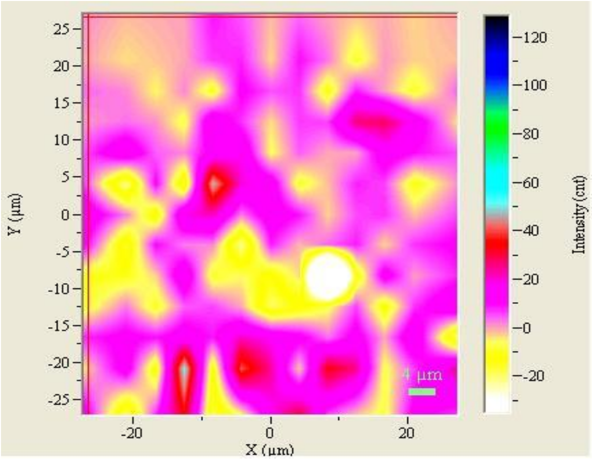

Figure S2. Surface distribution of D band intensity of PTG/P scaffolds

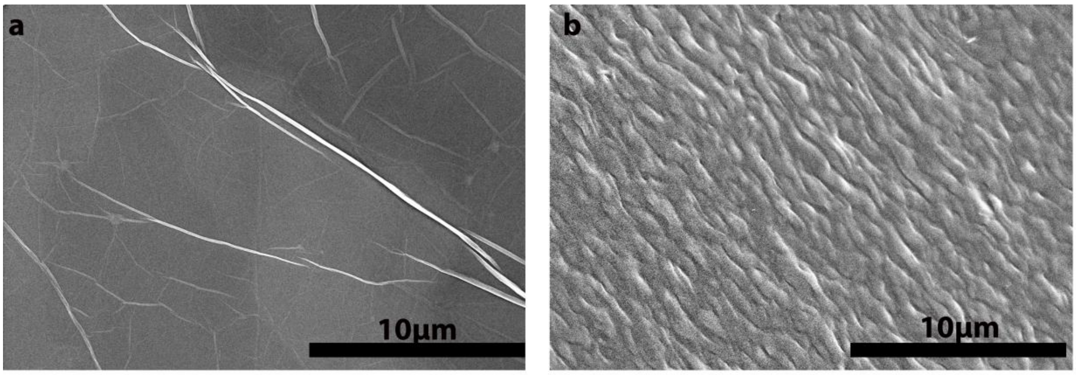

Figure S3. SEM images: (a) GO; (b) GO@peptide

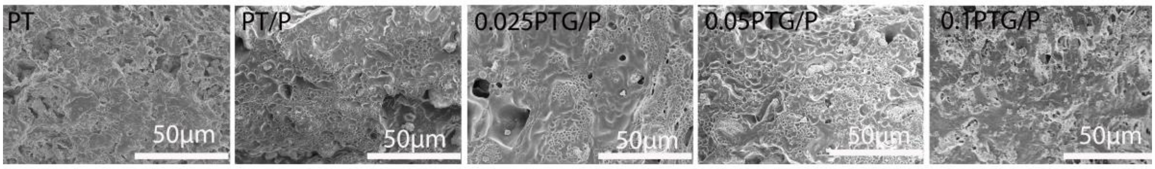

Figure S4 morphology of scaffolds after 8-week degradation

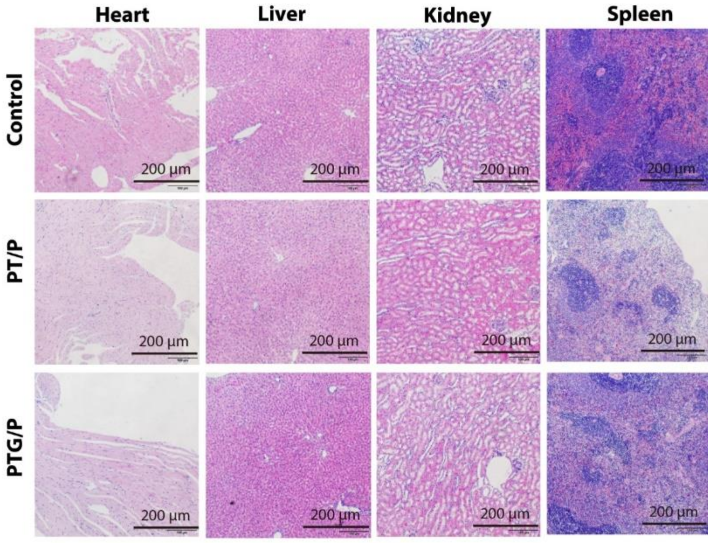

Figure S5 Histological examination of heart, liver, kidney, spleen with H&E staining
